# Mucin phenotypic expression and p53 gene abnormality of gastric super-minute well-differentiated adenocarcinoma: Re-evaluation with relationship between histogenesis of well-differentiated adenocarcinoma and intestinal metaplasia in distal stomach

**DOI:** 10.1186/1477-3163-4-14

**Published:** 2005-09-01

**Authors:** Ryo Wada, Toshikazu Yamaguchi, Takayuki Tanizaki

**Affiliations:** 1 The Department of Pathology, Juntendo Shizuoka Hospital of Juntendo University School of Medicine, Shizuoka, Japan; 2 The Department of Pathology(I), Juntendo University School of Medicine, Tokyo, Japan; 3 R & D Center, Biomedical Laboratories, Inc, Saitama, Japan

**Keywords:** Gastric differentiated adenocarcinoma, mucin, microsatellite, p53, MUC2, CD10, 45M1, MUC6

## Abstract

**Background:**

Although the gastric well-differentiated adenocarcinoma in the distal stomach has been thought to develop via a intestinal metaplasia-carcinoma sequence, there are some disproofs from new mucin examinations for minute-size lesions in same type carcinoma. The current study was performed and pointed out the new findings for the solution to the problem according to the point described above.

**Methods:**

12 super-minute lesions (less than 1 mm in maximum diameter) of well-differentiated adenocarcinoma in distal stomach (SMCa), which were detected from the pathological examinations of 210 surgically resected stomach specimens, and the mucosa adjacent to these carcinoma lesions, were examined by immunohistochemical mucin stainings (MUC2 and CD-10: intestinal phenotype, 45M1 and MUC6: gastric phenotype) and p53-overexpression. And the analyses of the replication error of the microsatellites in chromosome 17 related p53 gene (TP53 and D17S786) (RER-p53MS) were performed in SMCa lesions, adjacent mucosa to each lesion and other gastric mucosa with intestinal metaplasia, because all SMCa lesions showed p53-overexpression immunohistochemically, decribed below.

**Results:**

1. The carcinoma cells in all SMCa lesions were positive for 45M1 and p53. On the other hand, no positive carcinoma cells for MUC6 were seen although the pyloric glands and the remnant pyloric gland in the SMCa lesions in the same slides were positive for MUC6. Ten lesions (83%) had intestinal phenotypic mucin (10 lesions: MUC2 (+), 4 lesions: CD10 (+)). Two lesions (17%) were positive for only 45M1 (gastric phenotypic mucin). 2. All of the mucosa adjacent to SMCa showed intestinal metaplasia (complete type: 7 regions, incomplete type: 5 regions). 3. RER-p53MS was confirmed in 42% (5/12 regions) of SMCa, in 42% (5/12 regions) of the mucosa adjacent to SMCa and 14% (6/42 regions) of the other intestinal metaplasia mucosa.

**Conclusion:**

Most of the super-minute well-differentiated adenocarcinoma lesions in the distal stomach, which had both gastric and intestinal phenotypic mucin, are considered to develop from the tubular proliferative zone with the incomplete type of the intestinal metaplasia and p53 gene abnormality, while a part of them, which had only gastric phenotypic mucin, may derive from the gastric native tubules (non-metaplastic epithelium) with p53 gene abnormality.

## Background

Although the gastric differentiated adenocarcinoma has been thought to develop via a intestinal metaplasia-carcinoma sequence [1-4], there are some disproofs [5] that the gastric differentiated adenocarcinoma develops having a poor relationship to the intestinal metaplasia, from new mucin examinations for minute-size carcinoma [6,7] in same type. Thus, some problems for that histogenesis of the gastric differentiated adenocarcinomaare considered to be still remained.

To clarify the true histogenesis of digestive tract carcinoma, the extremely small-sized carcinoma lesions were found and these lesions including the mucosa adjacent to them must be examined pathologically and molecular-biologically. Because almost all lesions of the digestive tract carcinoma develop from the mucosal epithelium and the minute-sized carcinoma lesions and the mucosa adjacent to them may express the initial condition when each carcinoma develop.

According to the point described above, the current study was performed and pointed out the new findings for the solution to this problem.

In this study, firstly, the immunohistochemical mucin-stainings and p53-overexpression, and secondary, the replication error (RER) of the microsatellite (MS) in chromosome 17 related p53 gene of the super-minute sized differentiated adenocarcinoma lesions in the human stomachs, whose definition was described below, were investigated, because the carcinoma cells were positive p53 in all carcinoma lesions in the current study, and the new findings on histogenesis of gastric adenocarcinoma were revealed.

## Methods

**Figure 1:**
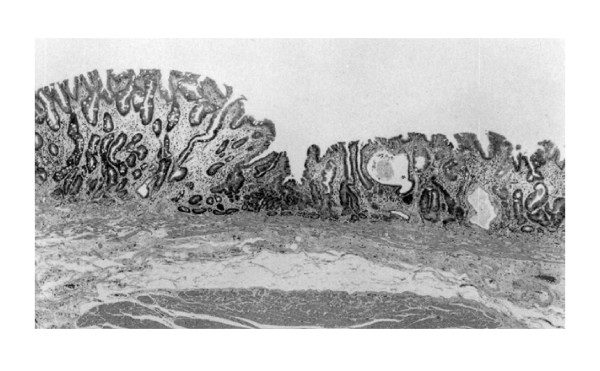
Typical case of SMCa. SMCa lesion is seen in the center region of this fugure. (×20, HE)

**Figure 2:**
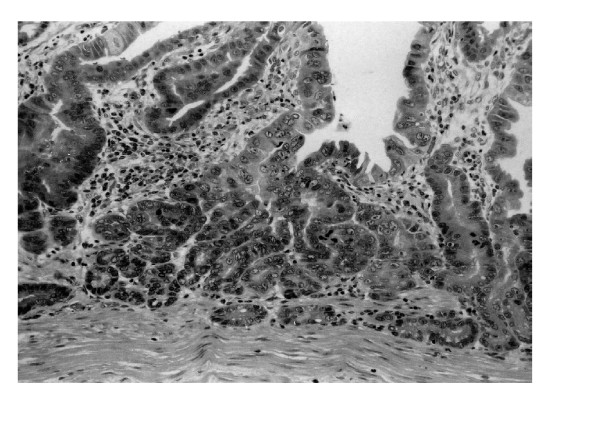
High power view of Figure 1 lesion. This lesion was diagnosed as high-grade intraepithelial neoplasia according to both the Vienna and WHO classification and as well-differentiated adenocarcinoma according to the classification of the Japanese Research Society for Gastric Cancer. (×200, HE)

Twelve tubular proliferative lesions (within 1 mm in maximum diameter) with cellular atypia and the boundaries to the neighborhood, which were diagnosed as high-grade intraepithelial neoplasia according to both the Vienna and WHO classification [8,9] and as well-differentiated adenocarcinoma according to the classification of the Japanese Research Society for Gastric Cancer [10] (the super minute-sized well-differentiated adenocarcinoma in the distal stomach: SMCa) (Figure 1 &2), were detected in the distal stomachs from the pathological examinations of 210 surgically resected stomach specimens with early cancer, assessed at the Department of Pathology, Juntendo University hospital and Shizuoka Hospital, between 1982 and 2003. Although in the cases between 1999 and 2003 the informed consent for pathological and molecular examinations were obtainted from each patient, in other cases the informed consent could not be obtained because the system of the informed consent for patients was built up at 1999.

The stomach specimens described above were fixed in 10% buffered formalin solution and prepared by cutting from almost the entirety of the stomachs area into 3 – 5 mm wide sections. Each section was embedded in paraffin and stained with hematoxylin and eosin (HE). And every sections with 12 SMCa lesions described above were cut serially and stained with HE, and the greatest dimensions of these lesions were determined (655 – 958 μm). The cases with SMCa lesions were considered as the cases of multiple gastric cancers by the criteria of Moertel et al. [11].

Using all paraffin blocks with SMCa lesions and 10 paraffin blocks with no neoplasia in each stomach, and the mucosa adjacent to SMCa lesions were examined by immunohistochemical mucin stainings as shown in Table 1 (MUC2 and CD-10: intestinal phenotype, 45M1 and MUC6: gastric phenotype). These 4 mucin stainings are more popular for detection the mucin phenotypes of the gastric differentiated adenocarcinoma [12].

**Table 1:** Immunohistochemical mucin antibodies

	**Commercial name**	**Positive cell**	**Dilution**	**Source**
**MUC2**	Human Muc2 glycoprotein	Goblet cell and their precursor cell	1:500	Novocastra, UK
**CD10**	CD10 glycoprotein	Enterocytes	1:200	Novocastra, UK
**45M1**	Peptide core of human gastric mucin (NCL-HGM-45M1)	Gastric surface mucous cell	1:100	Novocastra, UK
**MUC6**	Human Muc6 glycoprotein	Pyloric gland, mucous neck cell, cardiac gland, Brunner's gland	1:100	Novocastra, UK

And we have tried to examine a number of Exons for detecting the p53 gene abnormalities, because all lesions of the gastric super-minute well-differentiated adenocarcinomas showed overexpression of p53 immunohistochemically, described below (in "Results"). However, it was hard to examine it, because the target fici were too small and in post-formalin solution condition. Secondary, we have tried to estimate the existence or nonexistence of the p53 gene abnormalities using microsatellite markers in chromosome 17 related p53 gene (RER-p53MS).

### Immunohistochemical stainings

Immunohistochemical mucin-stainings were performed by the avidin-biotin-peroxidase-complex method with each condition as shown in Table 1. Recently, it has been pointed out that the data of the mucin phenotypes of the lesions will change accroding to what percentage of positive cells in each stainings is judged as the positive in each stainings [13]. In this study, the mucin phenotypes of the lesions were determined by positive (>0% of the carcinoma cells) or negative. Because the target lesions in this study were extremely small lesions, and even if only a carcinoma cell was positive, this positivity should be considered to be the effective and valuable data. And p53-oncoprotein antibody staining with microwave treatment was done in many sections (p53: DO-7, Novocastra Inc., UK).

### DNA extraction and analysis of RER-p53MS using microsatellite markers (TP53 and D17S786)

Paraffin blocks with the target foci mentioned above were prepared for DNA extraction. The target foci were microdissected using a 20-gauge needle, comparing the slide with HE staining in the same position. The extracted DNA was diluted with 500 μl DNA Isolation Solution (DNA isolater for DNA Extraction from Paraffin-embedded Tissue, Wako Inc., Osaka). In this study, target foci were SMCa and other gastric non-neoplastic mucosa and the extracted DNA from them were used as template DNA in polymerase chain reaction (PCR). However, in approximately half of target foci, DNA could not be extracted, because these target foci may be too small and moreover these materials were done by the formalin-solution. And decribed above, as the purpose of the molecular biological examinations was to know the existence or nonexistence of p53 gene abnormalities of target foci, TP53 and D17S786 were selected as microsatellite markers in the current study, according the proposition by National Cancer Institute Workshop [14].

**Figure 3:**
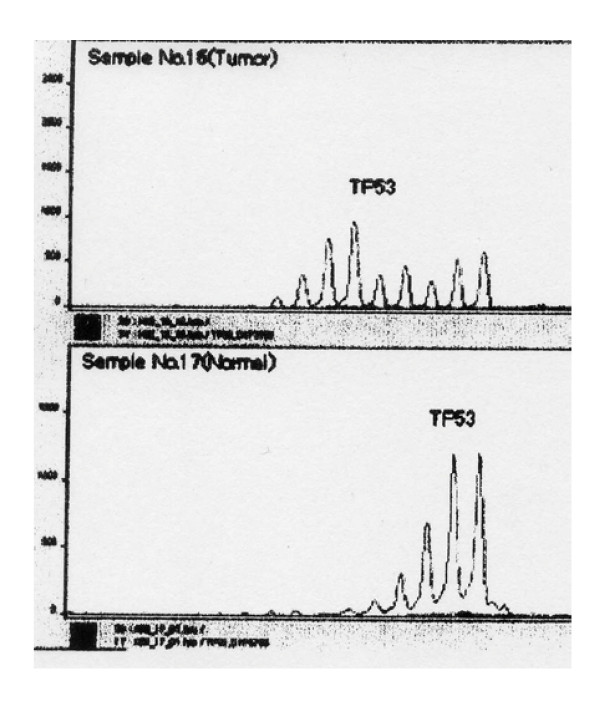
The shift (+) was determined when a new peak not recognized with non-neoplastic and metaplastic tissue (lower view in Figure 3) was confirmed with the target mucosa with intestinal metaplasia or SMCa lesions (upper view in Figure 3), that is to say, RER-p53MS was positive.

RER-p53MS was investigated using high resolution fluorescenced labeled PCR primers in proportion to the method of Tsuchida et al. [15]. The outline of it: 1. PCR was performed containing 1 μL of DNA lysate, 100 μM dNTP, 1.5 mM MgCl_2_, 1 μM each primer marked with fluorescent dye of three colors as blue, green and yellow, 0.625 U Taq DNA polymerase and 1 × PCR buffer [containing 10 mM Tris-HCl (pH 8.3 at 25°C), 50 mM KCl and 0.001%(w/v) gelatin] in a thermal cycler. 2. The electrophoresis was conducted 2 hours by means of an ABI-377 DNA auto-sequencer (PE Bio-systems, Inc., Foster City, CA, USA). 3. A comparison was made of peaks of same marker arising from gastric non-neoplastic and metaplastic mucosa and SMCa lesions, using the Gene Scan TM waveform analyzed softwave, and shift were assessed. The shift (+) was determined when a new peak not recognized with non-neoplastic and metaplastic tissue was confirmed with the target mucosa with intestinal metaplasia or SMCa lesions, that is to say, RER-p53MS was positive (Figure 3).

Although we have tried to examine the existence of Helicobacter pylori in the SMCa lesions and the mucosa adjacent to them, the surface mucous layer of specimen stomachs, in which Helicobacter pylori may often exist, was washed away at taking photograph of each specimen, and the relationship between SMCa and Helicobacter pylori could not be examined.

The data were analyzed statistically with Student's t-test (t-test) and chi-square test; a p-value of less than 0.05 was considered to be significant.

## Results

### 1. Mucin phenotypes of SMCa (Table 2)

**Table 2:** Mucin phenotypes of SMCa

**Case**	**MUC6**	**45M1**	**MUC2**	**CD10**
1	-	+	+	+
2	-	+	-	-
3	-	+	+	-
4	-	+	+	+
5	-	+	+	-
6	-	+	+	-
7	-	+	+	-
8	-	+	-	-
9	-	+	+	+
10	-	+	+	-
11	-	+	+	-
12	-	+	+	+

Total	0% (0/12)	100% (12/12)	83% (10/12)	34% (4/12)

**Figure 4:**
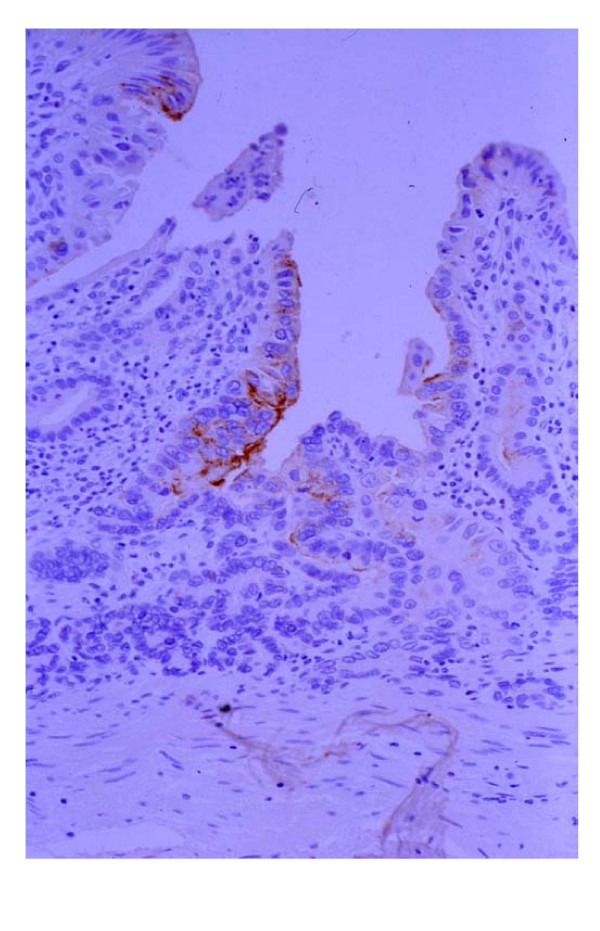
Among SMCa lesions, all lesions were positive for 45M1. (×200, 45M1 in Figure 1 lesion)

**Figure 5:**
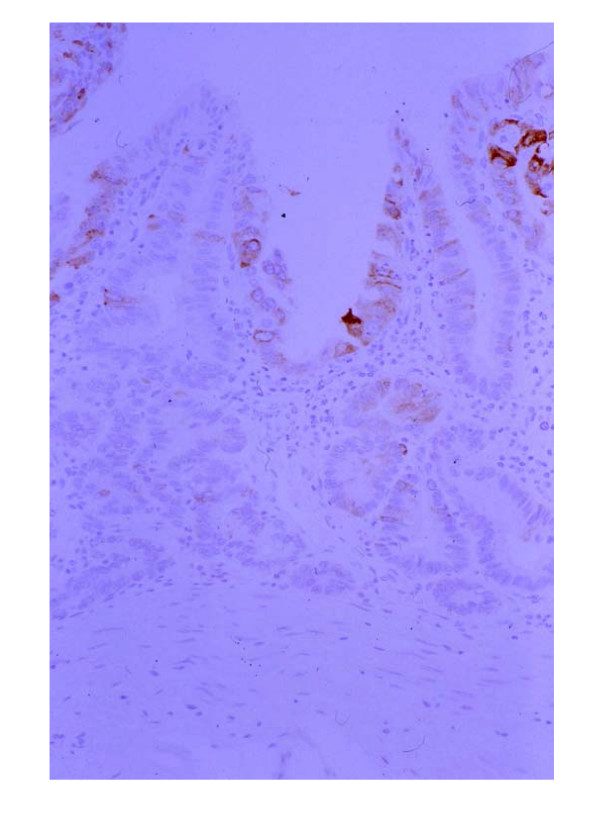
Ten lesions were positive for MUC2. (×200, MUC2 in Figure 1 lesion)

The carcinoma cells in all SMCa lesions were positive for 45M1 (Figure 4). On the other hand, no carcinoma cells in all SMCa lesions were positive for MUC6, although the pyloric glands and the remnant pyloric glands in SMCa lesions, which were seen in the same slides, were positive for MUC6. Ten lesions (83%) had intestinal phenotypic mucin (10 lesions: MUC2 (+), 4 lesions: CD10 (+)) (Figure 5). Two lesions (17%) were positive for only 45M1. That is to say, among SMCa lesions, 10 lesions (83%) had both gastric and intestinal phenotypic mucin and 2 lesions (17%) had only gastric phenotypic mucin.

### 2. The mucosa adjacent to SMCa lesions

All of the mucosa adjacent to SMCa showed intestinal metaplasia (complete type: 7 regions, incomplete type: 5 regions, by the criteria of Filipe et al. [16]).

### 3. p53-overexpression and frequency of RER-p53MS in SMCa and the mucosa adjacent to them

**Figure 6:**
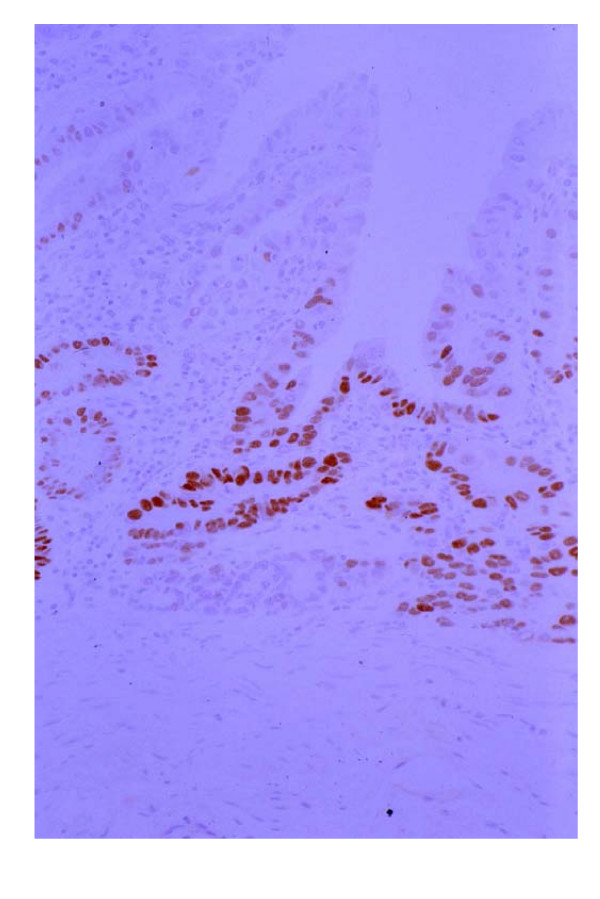
Anti-p53 antibody staining (p53) in Figure 1 lesion. Most of the carcinoma cells in this lesion showed a positive reaction for p53. (×200, p53)

Although most of the carcinoma cells in every SMCa was positive for p53 (Figure 6), there was no positive cell for p53 in the mucosa adjacent to SMCa and other intestinal metaplasia.

RER-p53MS was confirmed in 42% (5/12 regions) of SMCa, in 42% (5/12) of the mucosa adjacent to SMCa and 14% (6/42) of the other intestinal metaplasia mucosa (complete type: 17%, 4/23, incomplete type: 11%, 2/19), as shown in Table 3.

**Table 3:** Frequency of RER-p53MS in SMCa, the mucosa adjacent to them and intestinal metaplasia

	**TP53**	**D17S786**
SMCa	42% (5/12 resions)	8% (1/12 regions)
Mucosa adjacent to SMCa (intestinal metaplasia)	42% (5/12 regions)	17% (2/12 regions)
Other intestinal metaplasia	14% (6/42 regions)	5% (2/42 regions)

## Discussion

Although gastric differentiated adenocarcinoma is thought to develop from intestinal metaplasia [1-4], there are the disproof reports using a new mucin examination methods [5-7]. Thus, the histogenesis of the gastric differentiated adenocarcinoma may be still unclear.

The small carcinoma lesion is thought to be a good model for researching the histogenesis of digestive tract carcinoma, because almost all lesions of the digestive tract carcinoma develop from the mucosal epithelium and the minute-sized carcinoma lesions and the mucosa adjacent to them may express the initial condition when each carcinoma develop. Therefore, the carcinoma lesions must be as small as possible to examine the original findings in the mucin phenotypes or the gene abnormalities, and moreover the essential mucin expression in the small lesions must be determined as 'positive' even if a carcinoma cell was positive.

Thus, the mucin expression and gene abnormalities in super-minute-sized gastric well-differentiated adenocarcinoma, which have been shown in the initial stages of same type-carcinoma, are still thought to be unclear and the current study was performed focussing on this, and some interesting findings were revealed in the current study.

SMCa in the current study was defined as the tubular proliferation with cellular and structural atypia, diagnosed as high-grade intraepithelial neoplasia according to the Vienna [8] and WHO classification [9], and as well-differentiated adenocarcinoma according to the classification of the Japanese Research Society for Gastric Cancer [10]. Thus, SMCa lesions were thought to be the initial stage of development of the gastric well-differentiated adenocarcinoma.

Our results in the current study showed that 1. Among SMCa lesions, all carcinoma lesions were positive for 45M1 and p53, and no carcinoma cells were positive for MUC6. Ten SMCa lesions (83%) had intestinal phenotypic mucin (10 lesions: MUC2 (+), 4 lesions: CD10 (+)). Two SMCa lesions (17%) were positive for only 45M1. That is to say, among SMCa lesions, 10 lesions (83%) had both gastric and intestinal phenotypic mucin and 2 lesions (17%) had only gastric phenotypic mucin. 2. All of the mucosa adjacent to SMCa showed intestinal metaplasia (complete type: 7 regions, incomplete type: 5 regions). 3. RER-p53MS was confirmed in 42% (5/12 regions) of SMCa, in 42% (5/12 regions) of the mucosa adjacent to SMCa and 14% (6/42 regions) of the other intestinal metaplasia mucosa.

Although the mucin phenotypes of SMCa lesions differed from the data of other reports using the gastric minute differentiated adenocarcinoma [6,7], the reason of the differences may be caused the differences of the definition of the positivity in each stainings decribed above. In the current study, the lesions were judged as 'positive' even if a carcinoma cell was positive in each staining. The data in RER-p53MS of SMCa was almostly equal to the data of MS instability or the loss of heterozygosity in the gastric differentiated adenocarcinoma and intestinal metaplasia in other reports [17-19].

Focusing on the histogenesis of the gastric diffrentiated adenocarcinoma, the results of mucin expressions of SMCa revealed that gastric well-differentiated adenocarcinoma should not derive from the intestinal metaplasia with no remnant gastric original tubules (the tubules exchanged perfectly by intestinal metaplasia), because all of SMCa lesions had the gastric phenotypic mucin. However, most of SMCa may develop having a relashionship to the intestinal metaplasia, because they had also the intestinal phenotypic mucin. Namely, most of the SMCa lesions should derive from the gastric native tubule with a part of intestinal metaplasia and p53 gene abnormality.

On the other hand, a minority of SMCa had only gastric phenotypic mucin, are considered to develop from the gastric native tubules with no intestinal metaplasia and with p53 gene abnormality.

Thus, when the judgements of the mucin expressions in the gastric super-minute well-differentiated adenocarcinoma were strictly performed like as the current study, although the main route of the histogenesis of the gastric differentiated adenocarcinoma is thought to be the intestinal metaplasia-carcinoma sequence as before, it is very important that some well-differentiated adenocarcinoma may derive from the gastric native tubules with no relationship to the intestinal metaplasia. That is say, the intestinal metaplasia should not be the indispensable factor in the development of the well-ifferentiated adenocarcinoma in the distal stomach.

In the current study, the target lesions were limited to the lesions in the distal stomach, because the carcinoma lesions as like SMCa lesions could not be found in proximal stomach including the gastric cardia. Thus, we could not point out the histogenesis of the gastric well-differentiated adenocarcinoma in the whole stomach. The further study between the histogenesis of gastric differentiated adenocarcinoma and the intestinal metaplasia is expected.

## Abbreviations

SMCa, super-minute lesions (less than 1 mm in maximum diameter) of well-differentiated adenocarcinoma in distal stomach; RER, replication error; MS, microsatellite
